# Assistive technology: Occupational therapy assessment and services for people with dementia

**DOI:** 10.1177/03080226241252280

**Published:** 2024-05-21

**Authors:** Eleanor Curnow, Fiona Maclean, Brendan McCormack

**Affiliations:** 1 School of Health Sciences, Queen Margaret University, Edinburgh, UK; 2 Occupational Therapy and Allied Health & Social Care Science, Edinburgh Napier University, Edinburgh, UK; 3 Susan Wakil School of Nursing and Midwifery, Faculty of Medicine and Health, The University of Sydney, Sydney, NSW, Australia

**Keywords:** Assistive technology, occupational therapy, dementia, person-centred practice

## Abstract

**Introduction:**

Research suggests that services are not effectively providing suitable assistive technology for people with dementia. There is a need to understand the challenges facing practitioners to identify where service changes should be focussed to improve this situation.

**Method:**

This study used an online survey to explore the experiences of 41 occupational therapists working with people with dementia, and/or assistive technology. Eight participants subsequently agreed to participate in online discussion groups. Group discussions were transcribed and checked. Data responses from group discussions and open questions in the survey were analysed thematically using the person-centred framework to identify domains supporting or obstructing effective assistive technology service delivery.

**Results:**

Forty-one occupational therapist participants described challenges to providing person-centred assistive technology services. These included restricted access to assistive technology interventions, limited knowledge regarding developments in the field, variable funding, unsupportive systems, limited relevant training and difficulties working across health and social care sectors.

**Conclusion:**

There is a need to revise the systems surrounding the provision of assistive technology for people with dementia. Occupational therapists need access to training relative to this field, and systems need to be adapted to support the provision of person-centred care by widening access to assistive technology.

## Introduction

Assistive technology (AT) is an umbrella term for physical or digital assistive products and their related systems and services (World Health Organization and United Nations Children’s Fund (UNICEF), 2022). One purpose of AT is to offer strategies that can delimit the disabling influence of environments (Cook et al., 2020). Specifically, in dementia, AT has been promoted as a possible source of support, which can enhance safety, promote well-being and compensate for physical and cognitive deficits and maintain people living at home for longer (Brims and Oliver, 2018; Fleming and Sum, 2014).

As such, occupational therapists have been identified as having knowledge and skills in the assessment and provision of AT with people living with dementia (Boger et al., 2014), and evidence has highlighted the effectiveness of this professional contribution (Pappada et al., 2021). However, more recently, research has confirmed that an assistive technology and telecare (ATT) needs assessment followed by installation of indicated devices, and response services do not prolong the time people living with dementia can remain at home and are not cost-effective when compared with limited ATT (Howard et al., 2021). Suggested reasons for this include possible limitations in assessment, disregard for assessment recommendations, or AT deployment inconsistent with local service goals (Forsyth et al., 2019). Assessment limitations include narrow understanding of the aspects, which should be considered within a comprehensive assessment of a person with dementia or how these aspects relate to AT provision. Additionally, people with more severe dementia often have fewer assessment fields documented, perhaps because they cannot be reliably assessed by interview (Malinowsky et al., 2018), limited practitioner skills, or lack of assessment instruments validated for the assessment of people with greater cognitive impairment (Hansen et al., 2018; van Ooteghem et al., 2019). As some occupational therapists can only access a limited prespecified range of AT, assessment may focus on available interventions rather than the needs of the person with dementia (Gibson et al., 2016; Sugarhood et al., 2014; Ward et al., 2017).

While occupational therapists contribute to this area of specialism, knowledge gaps have been identified connected to the selection of AT for people living with dementia, including the adaptation of technology to meet individual needs (Sugarhood et al., 2014). It has been suggested that difficulties in accessing AT interventions can also arise from underfunding and confusion about the roles and responsibilities of different members of the community care team (Jarvis et al., 2017; Newton et al., 2016). Existing service arrangements for health and social care practitioners in the assessment and adaptation of AT with people, matched to their specific needs, is generally suboptimal, and calls for a user-centred approach to the delivery of AT have, so far, had little impact (Greenhalgh et al., 2015, 2016).

Overall, the provision of AT for people with dementia in the United Kingdom (UK) is highly fragmented with significant differences in access to services, service charges and product availability in different areas (Gibson et al., 2016). Health and social care generally provide only a limited range of AT products often based around community alarm systems, GPS monitoring, simple to use telephones, memory aids, or aids to assist with activities of daily living (Gibson et al., 2016).

In the UK, the Royal College of Occupational Therapists (RCOT, 2021) highlight that practitioners should deliver person-centred services, to enhance outcomes for service users, ensure better use of resources, decrease costs and increase satisfaction with care (Fatoye et al., 2022; Phelan et al., 2020). Person-centred services include and value user involvement in the choice and design of AT (Cook et al., 2020). Yet, how person-centred practice can be understood and applied in the context of AT interventions and occupational therapy practice in dementia services can remain opaque.

**Figure 1.**
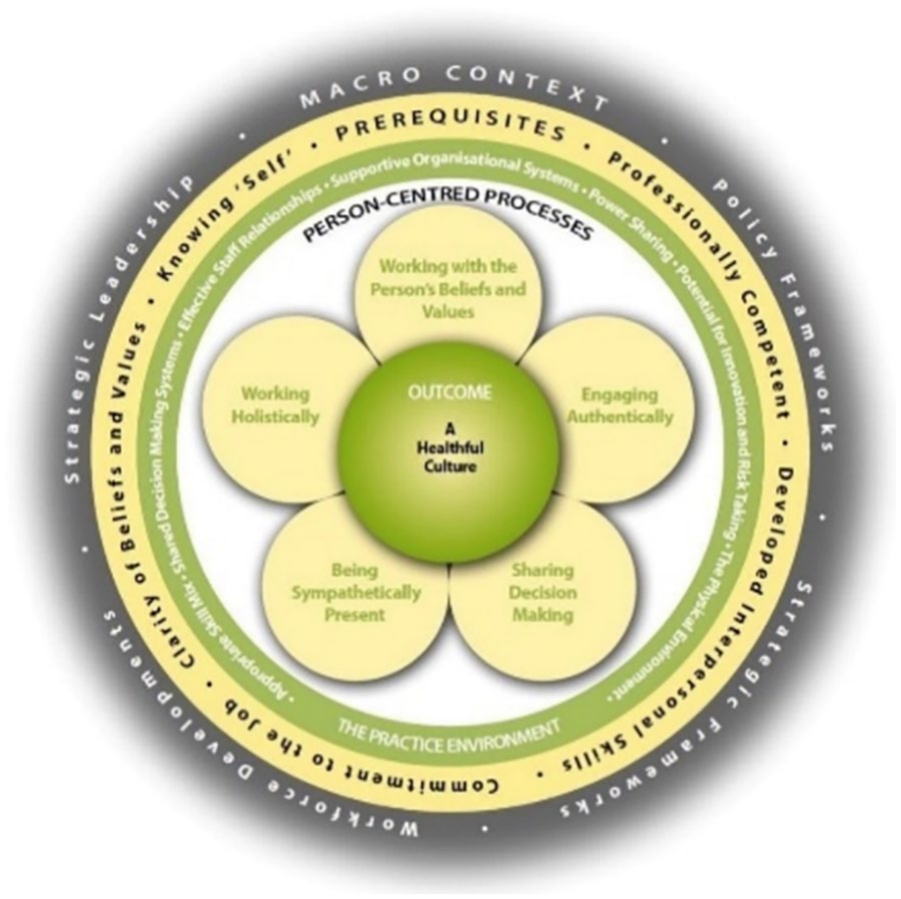
Person-Centred Practice Framework (McCance and McCormack, 2021). Reproduced with permission.

Person-centred practice is an approach based upon a therapeutic intent between professionals, patients and significant others, influenced by the wider context of organisational systems (McCance and McCormack, 2021). The person-centred practice framework (Figure 1) highlights key domains, which can offer a lens through which to operationalise and understand different influences on a person’s experiences of healthcare, as a mechanism to analyse current practice (McCance and McCormack, 2021). Consequently, this theoretical perspective guided the development of this research to explore in greater detail the complexities and extent to which AT services adopt, or are guided by, person-centred principles when working with people living with dementia who could benefit from AT.

Specifically, this study asks what supports or challenges the effective delivery of person-centred AT services for people living with dementia?

The study aims being, to:

1. Explore the experiences of occupational therapists who provide AT services, and/or work with people living with dementia.
2. Understand effective, person-centred AT services for people living with dementia.
3. Identify obstacles to the provision of person-centred AT services for people living with dementia.
4. Identify areas of good practice, including where enhancement can be achieved.

## Method

This study received ethical approval from the University Ethics Committee.

This study used mixed methods and was conducted in two stages. Stage 1 used an online survey to elicit data describing a diverse range of occupational therapist’s views and experiences of AT. Stage 2 involved conducting three discussion groups with eight occupational therapists who had completed the survey and consented to be contacted. Discussion groups were focussed on issues identified from the stage 1 survey responses to explore AT provision experiences more deeply (Barbour, 2001). Results were reviewed with an AT service provider to add further context. These stages will now be considered in detail.

### Stage 1: Online survey

The Joint Information Systems Committee (JISC) online survey was open from 29 November 2021 to 19 July 2022. The survey intended to explore the experiences of occupational therapists in AT provision for people with dementia and requested details of participant training, support and assessment tools. Survey items were based upon published research describing UK AT service provision (see Supplemental Materials; Forsyth et al., 2019; Gibson et al., 2016; Greenhalgh et al., 2015). Participant inclusion criteria were:

- Qualified as an occupational therapist, AND
- Involved in referring/ requesting, distributing, providing, or prescribing AT OR
- Has knowledge/ experience working with people living with dementia who continue to live at home, OR
- Has knowledge/experience in developing and/or researching AT for people with dementia; AND
- Who were able to read and respond to the survey in English.

The survey was developed by the research team and piloted by two HCPC-UK registered occupational therapists prior to distribution. Feedback from the pilot led to minor adjustments in the structure of the survey questions. Links to the survey were promoted by the research team on social media, including the RCOT Older People Specialist Section membership, and emailed to the Queen Margaret University Practice Educators and Alzheimer Scotland Allied Health Professions distributions lists. Participant consent was provided electronically.

Responses to open-ended survey questions were exported from JISC to excel. Responses were grouped according to the focus of the response then mapped to the person-centred practice framework (Figure 1), by the first author (EC) (Braun and Clarke, 2022).The analysis was discussed regularly with other researchers (FM, BM) to ensure that it was cohesive, meaningful and robust.

### Stage 2: Discussion groups

Survey participants were asked if they would be willing to participate in follow-up discussion groups. These groups aimed to explore issues raised during the survey to deepen understanding of context. Discussion questions were based on survey responses which elicited diverse views, or where there was a need for further clarification and explanation. Group discussions took place on MS Teams during May and June 2022 and were recorded then transcribed electronically. Participants and the group facilitator (EC) reviewed the transcripts for accuracy prior to analysis.

The first author read and re-read the data, then selected excerpts from the data and organised these into groups. Open coding was adopted to identify initial concepts, which were then grouped into categories. Categories were then verified, compared and collapsed within each other to form five identified themes. These themes were discussed with other members of the research team who contributed ideas and feedback. The first author read the data again to ensure that themes captured the essence of the data. This process was carried out by the first author, then discussed and refined through discussion with the second and third authors.

The themes were then mapped against the person-centred practice framework with a focus on understanding the extent to which service delivery of AT by occupational therapists was influenced by a person-centred approach.

### Stakeholder review

An iterative process of stakeholder review was incorporated throughout the development of this study. This included a person living with dementia and their caregiver, who reviewed the initial design of the study, providing feedback and comment. Their further planned involvement in the study was curtailed due to personal circumstance.

Following qualitative analysis, the identified themes were shared with a leading sales expert within the technology enabled care industry. Involvement in this process recognised awareness of the macro-context in which AT as a speciality exists in practice.

Feedback further confirmed elements of the themes identified throughout analysis.

## Results

### Participant characteristics

Forty-one participants completed the online survey, of which eight continued to participate in the online discussion groups. The participant characteristics are outlined in Table 1.

**Table 1.** Participant Characteristics.

Participant Characteristic	Online survey (*N* = 41)		Discussion group (*N* = 8)	
Role*	*n*	%	*n*	%
I supply/ distribute (or previously supplied/distributed) Assistive technology	4	10		
I refer (or have previous experience of referring) people for possible use of assistive technology	28	70	4	50
I am/have been involved in assessment, prescription, monitoring, and follow-up of people using assistive technology	15	37.5	4	50
I am/have been involved in assessment, prescription, monitoring and follow-up of people living with dementia	23	57.5	4	50
I am/have been employed as an occupational therapist	33	82.5	5	62.5
How long have you been qualified as an Occupational Therapist?				
0–5 years	4	9.8		
6–10 years	4	9.8		
11–15 years	11	26.8	1	12.5
16–20 years	6	14.6	3	37.5
21–25 years	8	19.5	3	37.5
26–30 years	5	12.2		
31–35 years	2	4.9		
36–40 years	1	2.4		
Not known			1	12.5
In which region/country do you work?				
Scotland	25	61.0	5	62.5
England	10	24.4	2	25
Wales	2	4.9		
Ireland	2	4.9		
Other	2	4.9	1	12.5
Employer*				
NHS	29	70.7	4	50
Local Authority/Council	7	17.1	5	62.5
Self employed	1	2.4		
Retired	1	2.4		
Research	1	2.4		
Private Organisation	2	4.9		
Grade				
Council/LA	4	9.8	2	25
Band 5	3	7.3		
Band 6/Senior Practitioner	17	41.5	4	50
Band 7	9	22.0		
Band 8/8a	3	7.3	1	12.5
Not known	5	12.2	1	12.5
Practice setting*				
Community/Primary care	25	61.0	4	50
Hospital/Inpatient/ward/acute	16	39.0	2	25
Equipment/aids/adaptations/assistive technology	4	9.8	3	37.5
Social work	3	7.3		
Elderly/older adult/frailty	12	29.3		
Dementia/organic	6	14.6	1	12.5
Mental health	8	19.5	1	12.5
Learning disability	1	2.4		
Stroke/Neurological	2	4.9	1	12.5
Research	2	4.9		
Rehabilitation	3	7.3	1	12.5
Disability	2	4.9		
Orthopaedics	1	2.4		
Visual Impairment	1	2.4		

* Participants were able to select more than one answer.

### Key questionnaire results

Participants survey responses indicated that they used a range of assessments in practice relative to provision of AT equipment, including AMPS (*n* = 9, 21.9%), COPM (*n* = 4, 9.8%), Claudia Allen/ LACLS (*n* = 15, 36.6%) MOHOST (*n* = 8, 19.5%), ACE (*n* = 12, 29.2%) and non-standardised assessments (*n* = 16, 39.0%). Participants found assessment tools were limited by sensory, language and communication differences and time.

Most survey respondents (*n* = 27, 65.8%) felt that they were able to access a range of AT interventions, which met the needs of people with dementia; however, only five respondents (21.8%) had unrestricted access to AT they were able to consider in practice. Five respondents (12.8%) could only make a generic referral for AT services. Participants targeted risk reduction (*n* = 20, 48.8%), and priorities identified by the person with dementia (*n* = 13, 31.7%) over caregiver priorities (*n* = 2, 4.9%) or autonomy and independence (*n* = 3, 7.3%). Most respondents (*n* = 33, 80.5%) felt that there were times where AT may be appropriate but was not considered. Participants reported that this was due to restrictions related to funding, internet access, limited time prior to discharge and/or absence of a caregiver to respond to alerts. Respondents enhanced their knowledge in this field through a variety of continuing professional development (CPD) activities, informal training, conversations with colleagues, taking time to explore options, online resources such as web pages via Alzheimer Scotland, and through using case study discussions. Thirty-five (85.4%) respondents felt that there was a need for additional support to improve the effectiveness of AT services. Suggestions for improvement included additional training, and closer working relationships between the different services involved in AT provision.

## Discussion group results

Analysis of the transcribed discussion group conversations identified five themes: (1) system structure challenges facing Occupational Therapists providing AT; (2) restricted range of AT; (3) role of family carer and implications for AT needs assessment and provision; (4) CPD needs; and (5) need to avoid restricted, supply-led approaches. Each theme will now be discussed in turn.

### System structure challenges facing Occupational Therapists providing AT

Participants identified several obstacles which limited the provision of AT matched to individual need. Unsupportive organisational systems, poor communication and complex processes restricted person-centred practice when working across different institutions and organisations. One participant described how access to AT was restricted to social care employees:

If it’s a hospital occupational therapist and it’s our area, they cannot [order AT], they have to request that, and it has to be a social care member of staff whether it’s an OT or an OT assistant or social care staff. They would then take that forward to look at that equipment.[P3]

This created a divide between the occupational therapist with expertise in working with people with dementia, and the person providing the AT, which could result in duplication of assessment for the person with dementia. It is possible that this also meant identified needs were not met where information was not successfully transmitted between organisations.

So, I can assess someone in hospital and say they need a falls detector or a bed occupancy sensor, but then I have no way of following up if that’s still required once they’re home or if they need something that’s a bit more sophisticated.[P5]

there’s no system as far as I’m aware where we can follow-up technology that’s been delivered and how it matches and how it works for the individual.[P5]

### Restricted range of available AT

Participants reported restrictions limiting which AT they could use.

We have criteria where we can’t access certain technology because [. . .], that person doesn’t fit a particular diagnosis.[P4]

Available AT was mostly focussed on a narrow range of needs often related to the reduction of risk rather than enhancing quality of life.

it tends to be things around safety or communicating with others, safe walking or controlling their environment. So, we have to kind of just have a small focus really.[P7]

AT was often not actually used following installation, suggesting that it was not meeting the needs of the individual.

In the community we came across a statistic where something like 90% of OTs provided a CAS [Community Alarm System] alarm. We think that the actual statistic of people using the CAS alarm was about 40%.[P6]

which may be partly due to the desire of occupational therapists to feel that they were able to offer some sort of intervention to support people with dementia even if they were aware this was not ideal.

I guess the challenge there is really identifying if telecare is the right thing for someone and are we assigning telecare for the right reasons or are we doing it because we’ve got some perceived risk as an OT, and we can’t provide the all ‘singing and dancing’ package that we would like but it somehow makes us feel safe by giving somebody an alarm[P6]

### Role of family carer and implications for AT needs assessment and provision

Participants reported that at times they found it difficult to provide AT which met the needs of both the person with dementia and their caregiver. Responses indicated that in some cases, family members requested AT to reassure them rather than to assist the person with dementia.

Family want peace of mind and they want technology for their peace of mind, even though it’s not actually in the person’s best interests. [P7]

While often people with dementia rely on informal caregivers to provide care to prevent institutionalisation, the importance to balance the needs and wishes of both parties was recognised.

People with dementia need to be part of the conversation[P6].

In addition to AT provided to meet caregiver needs rather than the needs of the person with dementia, AT can often rely on caregiver input. Caregivers may be required to respond to alarms, maintain devices including replacing batteries and can influence the acceptance of AT by their relatives. Further, as most AT is provided to reduce safety concerns rather than improve quality of life, this may also mean that fewer people with dementia see this as a priority.

Telecare is used least for people living on their own.[P6]

Participant occupational therapists also suggested that earlier identification of people with dementia could enhance provision of AT for this population.

### Continuing professional development

Participants reported difficulties in becoming professionally competent due to problems accessing training. In some cases, training was restricted to a short single session, which introduced AT available within their locality. Some participants were based within specialist teams with expertise in AT and were able to develop training sessions, which suited their needs.

We link with companies, and they do training on their own equipment.[P4]

Participants with a particular interest in this field enhanced their knowledge through personal development activities.

I would buy things and test them myself [P2]

Any knowledge and training you have tends to be self-learned [P5]

Although time for such personal research into an ideal intervention was restricted in some cases by pressure to discharge people with dementia home from hospital,

it would be amazing to spend more time looking at digital devices to enable rehab. But actually, there was a pressure to get somebody out of hospital.[P5]

In addition to training, participants reported difficulties accessing information regarding AT for specific situations, which restricted their ability to identify the most appropriate intervention for people with dementia.

I’d quite like to see something that’s really accessible on, you know, RCOTs website that you can just go in, you can see the latest technology, you could look at case studies of what’s been used before.[P8]

You know you want to be able to actually see things, try things out to then be able to explain to families and patients exactly what it is.[P6]

### Need to avoid restricted, supply-led approaches

Participants working in centres of specialist AT practice identified the ideal process of providing AT and how this aligns with person-centred practice.

it’s all about your function, your assessment around that and then matching technology just like you would any other piece of equipment.[P6]

However, there was recognition that in some cases this was not what was happening.

I notice here with colleagues very much try to automatically fit technology to the person, so if someone is purposefully walking, they’ll think right, they need a GPS tracker but don’t actually consider why they’re walking in the first place, and actually that’s the crux of the assessment is why? Why are you going out?[P2]

The restricted range of available AT meant that one person could only be offered a fall detector or GPS tracking following a fall rather than AT, which could have promoted access to entertainment within her home.

After a telephone call with her daughter and asking the right questions, it came to light that in losing her eyesight she was not able to read or watch TV and when her hearing deteriorated, she was not able to listen to the radio or music so she would walk up and down the High Street and this is where she fell.[S6]

Provision of AT was also complicated by issues with funding AT purchases, and aftercare such as maintenance of the device.

There’s so many factors within that that one-to-one assessment as we know, as occupational therapists. But then you kind of get into the depths of it in terms of who purchases it, who maintains it, who’s then owning it and it kind of leads to a kind of a bit of a ripple effect really from that point of view.[P7]

a big difference [to what AT is available] is maybe around funding and provision[P4]

### View of AT provider

The AT service provider confirmed that purchases of technology were often made in response to budget pressures rather than in response to individual needs. For example, purchases were made at the end of the financial year when it became clear that there were sufficient funds to make a purchase. Additionally, it was their view that devices were sometimes purchased when leasing should have been the preferred option. This means that the purchaser is not committed to the ongoing costs associated with leasing items, but also that devices were not repaired or updated throughout the term of the lease.

The AT service provider was unaware of training courses relating to AT provision and adaptation and felt that there was a need in this area.

Identified themes were aligned with the person-centred practice framework (Figure 1). Findings from the research will now be discussed in relation to the domains of this framework: macro-context, prerequisites, the practice environment and person-centred processes (McCance and McCormack, 2021).

## Discussion and implications

This study surveyed occupational therapists’ knowledge, understanding, role and contribution to AT in the field of dementia through an online survey and online discussion. Findings indicate that while respondents prioritised person-centredness in theory, in practice, organisational systems and processes restrict the provision of individualised AT interventions for people with dementia. The findings inform further research in the field and have relevance for policy and practice.

Previous research has indicated that barriers to technology adoption by occupational therapists include affordability, time and increased awareness, education, and training (Mcgrath et al., 2017). Similar concerns are noted here; however, wider issues were also revealed connected to the provision of AT interventions, which hinder person-centred practice. Specifically, existing organisational systems in which occupational therapists are situated can limit the potential impact of professional contribution. Poor communication between health and social care services restricts sharing of knowledge and expertise, complex funding models limit availability of AT according to diagnosis and procurement processes designed to control expenditure are examples. More widely, these systems differ from region to region, nation to nation, further adding to professional confusion. While there are occasional specialist services, typically AT is provided by a diverse range of professionals in existing health and social care roles, who do not possess dedicated skills or knowledge in this field. This represents a barrier to building and developing a critical mass of expertise to influence provision of AT, and the small numbers of staff involved mean that success can be restricted by loss of key team members (Sugarhood et al., 2014).

When considering person-centred processes, working with a person’s beliefs and values are key. In occupational therapy, a core role of the profession is to support people to perform their desired occupations (Mcgrath et al., 2017), and there is a need for therapists to explore the potential of AT to promote meaningful lives (Goodall et al., 2021). However, findings from this research indicate that AT interventions are often provided to support caregiving roles and responsibilities or relieve caregiver stress through risk reduction rather than supporting participation or promoting quality of life for the person living with dementia (Schepens Niemiec et al., 2022). This may also partly explain why AT is provided less frequently for people living alone (Curnow et al., 2021).

As AT has been strongly associated with safety, it is often not considered, or available, in relation to different needs (Brims and Oliver, 2018; Evans et al., 2015). It is not known if previous experience has taught occupational therapists to focus their assessment on needs, which can be met by available services, or if this is a response to a person’s assessed need (Hansen et al., 2018). However, there is scope for AT to be used in more imaginative ways to meet in response to the needs of people with dementia, which would in turn reduce the occurrence of adverse outcomes (Greenhalgh et al., 2016; Sugarhood et al., 2014). In practice, however, the limited choices available within AT services are likely to confound occupational therapists who hope to support meaningful activities for individual needs (Hansen et al., 2018; Puaschitz et al., 2021). Consequently, a lack of awareness of AT leaves healthcare professionals unable to consider options, reducing the possibility of onward referral to AT services (Greenhalgh et al., 2015; Jarvis et al., 2017).

The macro-context influencing person-centred practice is key, including workforce development. Yet, encouraging and promoting wider professional development of knowledge and understanding connected to the potential of AT in partnership with people living with dementia is hindered by the lack of educational opportunities in this field. Findings from this study indicate that there is a haphazard approach to professional development, with respondents highlighting the dearth of available ongoing CPD opportunities. Where access to training was available, this tended to be provided ‘in-house’, re-affirming existing provision, rather than recognising wider potential. There remains a need for partnership education across health and social care services, de-limiting access for occupational therapists situated in organisations where access to AT was via onward referral. To enhance professional competence in this field, there is a need for undergraduate and ongoing postgraduate education in the provision and individualisation of AT. Particularly, there is a need for accessible resources, which provide regular updates on available AT, and examples of how AT has been used successfully with people with dementia (Gibson et al., 2016; Howard et al., 2022).

The macro-context in which health and social care services operate needs to be transformed to be able to deliver person-centred care focussed on prevention and early intervention (Health Education England, 2017). The majority of AT services in the UK are provided by local authorities (Gibson et al., 2016). However, where social care expenditure relating to AT results in cost savings only within healthcare services, there is little incentive for local authorities to increase service provision (Sugarhood et al., 2014). This restricts the provision of AT by occupational therapists or other professionals working within the National Health Service (NHS). This divide between the roles of health and social care professionals reduces opportunities for follow-up support, which accounts for changes in the health and functioning of the person with dementia and how their need could be met through AT (Howard et al., 2022).

When considering the contribution of occupational therapy with people living with dementia and the provision of AT, the findings of this study illustrate a need to critically consider the influence of wider socio-political factors, influencing existing service delivery. While the findings here reveal limitations in the way in which therapists can work with a person with dementia and their needs connected to AT in practice, more broadly these are because of barriers at the level of macro-context. It is important that strategic policy is developed in a way which is informed by the voices of lived experience, to enable ambitions and timelines that can improve population well-being (Baldie et al., 2021).

There is therefore a need to re-design AT services alongside and informed by the experiences of people living with dementia. To allow the provision of AT to meet the unique needs of people living with dementia, commissioning models should move from a standardised approach to consider personalised interventions (Greenhalgh et al., 2015). Current provision restricts AT services to a limited range of AT devices from a small number of manufactures, and stand-alone products are rarely available (Gibson et al., 2016). There is also a need for increased focus upon AT supporting everyday activities and quality of life of people with dementia in addition to existing safety and caregiver support (Gibson et al., 2016). Variation in pricing structures and funding depending on diagnostic criteria, geographical location or type of AT together reduce equality in service provision (Gibson et al., 2016; World Health Organization, 2023). This is compounded by rules requiring people with dementia and their families to personally meet the costs of renting AT devices or purchasing additional services such as emergency response (World Health Organization, 2023).

While strategic leadership is recognised as a core foundation of person-centred cultures (Baldie et al, 2021), the lack of educational opportunities for the profession of occupational therapy connected to AT and dementia is of concern. For innovation to thrive, there is a need not only to invest in developing knowledge and skills, but also to create an environment where this can happen. To allow people closest to practice the autonomy to engage with service users, to ensure shared decisions that are right for them, strategic leadership needs to evolve to allow this to happen.

Overall, there is a need to review the services which are responsible for the provision of AT for people with dementia to reduce the barriers to person-centred care. By examining barriers to effective AT provision through a person-centred lens, this study provides information on areas which should be targeted for improvement in practice.

### Limitations

Participant recruitment was lower than anticipated despite multiple methods adopted to promote recruitment. Convenience sampling may mean that those who participated had a particular interest in this field, and therefore, their views may not be representative of all occupational therapists. It was intended to include a co-researcher with lived experience of dementia across all stages of this study, but this was not possible due to personal circumstances, and they withdrew following development of the grant application.

## Conclusion

This study explored the views and experiences of occupational therapists who provide AT and/or work with people with dementia using online survey and online discussion methods. While occupational therapists reported benefits associated with the provision of AT for people with dementia, participants also noted that AT provision was often restricted by limitations including access to training and related resources, knowledge regarding the individualisation of AT, access to AT interventions, complications regarding criteria for funding and provision and complex systems surrounding AT referral between organisations. There is, therefore, a need for AT services to be re-considered to ensure that organisational systems are in place that aim to support occupational therapists to meet the unique needs of individuals with dementia. By extension, to enable person-centred cultures of practice that can enable collaborative decision-making in partnership with people living with dementia and to support the autonomy of therapists working in health and social care.

Ongoing CPD, including postgraduate educational opportunities, is needed to continue to grow enhanced confidence, knowledge and awareness of the complexity of this specialist field of practice. More broadly, access to bespoke education would advance and further define the importance of creating wider occupational therapy integrated leadership roles in AT, with the potential to bridge across systems of health and social care. This would thereby increase the likelihood of equality of access to service provision, while also supporting the evolution of collaborative decision-making, including those with lived experience, to set the strategic direction of AT services of the future.

**Key findings**

- Occupational therapists require ongoing education to enhance expertise in AT provision for people with dementia.
- Organisational systems should be transformed to enable a person-centred approach to the provision of AT.

**What this study has added**

Occupational therapists working with AT and people with dementia reported difficulties providing person-centred care due to limitations in education, availability of AT, inter-organisational barriers and issues with models of funding.

## Supplemental Material

sj-docx-1-bjo-10.1177_03080226241252280 – Supplemental material for Assistive technology: Occupational therapy assessment and services for people with dementia Supplemental material, sj-docx-1-bjo-10.1177_03080226241252280 for Assistive technology: Occupational therapy assessment and services for people with dementia by Eleanor Curnow, Fiona Maclean and Brendan McCormack in British Journal of Occupational Therapy

sj-pdf-2-bjo-10.1177_03080226241252280 – Supplemental material for Assistive technology: Occupational therapy assessment and services for people with dementia Supplemental material, sj-pdf-2-bjo-10.1177_03080226241252280 for Assistive technology: Occupational therapy assessment and services for people with dementia by Eleanor Curnow, Fiona Maclean and Brendan McCormack in British Journal of Occupational Therapy
