# Supplementary material for: Assistive technology: Occupational therapy assessment and services for people with dementia
Source: Br J Occup Ther. 2024 May 21;87(9):564–73. doi: 10.1177/03080226241252280 (PMC11887907; doi:10.1177/03080226241252280)
Supplement: sj-docx-1-bjo-10.1177_03080226241252280 – Supplemental material for Assistive technology: Occupational therapy assessment and services for people with dementia [file sj-docx-1-bjo-10.1177_03080226241252280.docx]

Focus Group/ Group Interviews

Introduce selves

Brief introduction to study: RCOT Funded study to explore occupational therapists experience of providing AT for pwd. This is the second stage of the study following online survey. In this session we hope to explore effective practice in this field. We are running three groups and will be including OTs from different workplaces with different levels of experience. All views are important.

Ethics: We will be recording/ transcribing this session. Following check of the transcription – recording will be deleted. Transcription will be anonymised. Any questions/ concerns?

**I will start recording now.**

Ask participants to introduce themselves – sharing information they are happy to share regarding workplace, and experience of AT provision/ dementia.

1. Understanding of Assistive technology

- Digital v standard equipment – not telecare?

2. Confidence in assessment: Survey responses indicate OTs undertake

- To what extent?
- What does this tell us?
- Paradox of referral – what is included in referral?
- What does this feel like for the person?

3. Influencers on Practice

- Training is often inhouse – how is this? Quality? Content?
- Availability/ accessibility of AT
- To what extent does person-centred approach influence assessment/ referral/ recommendations? Where does risk come into this?
- What can be enhanced?
