# Supplementary material for: Assistive technology: Occupational therapy assessment and services for people with dementia
Source: Br J Occup Ther. 2024 May 21;87(9):564–73. doi: 10.1177/03080226241252280 (PMC11887907; doi:10.1177/03080226241252280)
Supplement: sj-pdf-2-bjo-10.1177_03080226241252280 – Supplemental material for Assistive technology: Occupational therapy assessment and services for people with dementia [file sj-pdf-2-bjo-10.1177_03080226241252280.pdf]

AT Needs Assessment for People with Dementia

Study Information

We are inviting occupational therapists to take part in an online study about the process of assistive technology assessment for effective delivery of services for people living with dementia at home.

This study is being conducted by a team of researchers based at Queen Margaret University, Edinburgh.

We are investigating the optimal assessment processes which support people with dementia to receive the assistive technology they require. This is important to reduce unmet need for people with dementia.

The study has been funded by the Royal College of Occupational Therapists Research Foundation.

This study builds on previous research which identified:

1. Limited evidence for the effectiveness of assistive technology supporting people with dementia to remain at home; and
2. A requirement to increase knowledge and skills relating to the assessment of assistive technology needs.

We now need to find out what expert clinicians know about assessment and provision of assistive technology. This will help us to understand what works well, and where there are opportunities for enhancement.

Assistive technology is defined by Alzheimer’s Society (2019) as devices or systems which support a person to maintain or improve their independence, safety and wellbeing. It includes a wide range of items from electronic pill boxes which remind you to take

medication, to 'smart home systems' which can adjust heating or lighting.

We welcome your participation in the online survey if you have experience / expertise as someone:

- who is an occupational therapist; and
- has been involved in referring/ requesting, distributing, providing, or prescribing Assistive Technology, OR
- has knowledge/ experience working with people with dementia living at home; OR
- has knowledge/ experience in developing and/or researching Assistive Technology for People with dementia,
- who is aged 18 or over and feels able to read and respond to this survey in English.

How does the study work?

This is a research study, so it is important that you know what is involved. After reading the information below, if you wish to learn more, please contact us at the email address provided.

You will be asked to fill an online survey, and you need to complete this on a laptop or computer.

In each survey you will be asked to:

- provide us with some information about you,
- provide answers to questions relating to clinical practice assessing the needs of people with dementia, and the selection of appropriate interventions.
- tell us whether you think we have missed anything that is important to practice in this field.

This study will probably take you around 30-45 minutes to complete.

Ideally you would complete the survey in one session but you can save your answers and come back later to complete it. Select ‘finish later’ at the foot of the screen if you need to do this.

We will use people’s responses to develop recommendations for further research in this area, and to identify requirements for resources or training which will support practice.

Each survey is completed anonymously and we will keep the results confidential and store them securely for ten years. We don’t think you will find any questions upsetting.

You do not have to take part. If you wish to, we will ask you to read the consent

information below. If you are happy to continue, click on the option to say you consent to participating in the study and that you have received information about it in an appropriate way. You can stop at any time without giving a reason by clicking the x at the top of the screen to exit the survey. Please note that you have the right not to answer any question without providing a reason for your decision. When you click on ‘submit’ at the end of the survey, your responses will become part of our anonymous database.

What happens next?

This study may not benefit you directly, but we hope that it will help us work towards increasing the quality and equity of Assistive Technology service provision. We will store the survey responses in a password protected server space for ten years after we have finished the survey and will write this up for a research journal and use it to inform further work. You will not be identifiable in any reports.

Who is involved in this survey?

This survey is being carried out by a research team which includes academic staff from Queen Margaret University. The study has been reviewed by an ethics committee in Queen Margaret University Edinburgh and given a favourable ethical opinion.

Contact details:

I am the Principal Investigator and am coordinating the activities within the research project. Please contact me if you have any questions or would like to learn more about the project:

Eleanor Curnow

Queen Margaret University, School of Health Sciences, Musselburgh, EH21 6UU
Tel: 0131 4740000 – *phone this number and ask for me by name. If this is proving difficult, ask for 'Operator', then ask the person who answers for me*.
Email: ecurnow@qmu.ac.uk

This survey is GDPR compliant. You can access GDPR information by indicating "yes" below.

- ○ Yes
- ○ No

GDPR Compliance

The type of personal information we collect:

- Personal identifiers, contacts and characteristics (for example, name and contact details)

How we get the personal information and why we have it:

Most of the personal information we process is provided to us directly by you for one of the following reasons:

- to contact you about research in which you have expressed an interest
- in order to carry out research

We use the information that you have given us in order to:

- contact you about our research
- carry out research

Under the General Data Protection Regulation (GDPR), the lawful basis we rely on for processing this information are:

- We need it to perform a public task.

Your information is securely stored online in the EU.

We keep your contact information only for as long as is necessary for the purposes described above, and not for longer than twelve months.

Data Protection | Queen Margaret University (qmu.ac.uk)

Consent

Many thanks for your interest in this study, your participation is valued. By participating in this online survey, you are agreeing to the following statements:

- I confirm that I have read and understand the participant information for this survey.
- I confirm that I have been provided with the option to contact the research team for more information should I wish to.
- I understand that my participation is both voluntary and anonymous and that I am free to withdraw at any time without giving any reason, without my legal rights being affected.
- I understand that relevant sections of the data collected during the study may be looked at, and audited, by individuals from the Sponsor (Queen Margaret University).
- I understand that collected data, may be used in an anonymised form to inform academic publications or conference presentations.
- I may decline to answer any of the survey questions without providing a reason for my decision.
- I understand that once I have submitted my responses to this anonymous online survey, it will not be possible to withdraw my answers.
- I agree to my anonymised data being used in future studies.
- I confirm that I am at least 18 years of age.
- I agree to take part in the study.

Having read the above

○ I agree with the above statements and wish to participate in this study

○ I disagree with one or more of the above statements and do not with to participate in this study

Brief Guidance on completing the survey

This survey will probably take you around 30-45 minutes to complete.

We are keen to understand your views of the issues raised in this survey. Please use the comments boxes to provide any additional detail that you feel might be helpful.

Ideally you would complete the survey in one session but you can save your answers and come back later to complete it. Select ‘finish later’ at the foot of the screen if you need to do this.

If you have any questions about completing the survey, please contact one of the researchers on the project – Eleanor Curnow ecurnow@qmu.ac.uk - and she will help you.

Section 1: We are interested in finding out about you, to help us when analysing the results

Which of the following descriptions applies to you (please tick all that apply)?

- ☐ I supply/ distribute (or have previous experience of) Assistive Technology
- ☐ I refer (or have previous experience of) people for possible use of Assistive Technology
- ☐ I am/ have been involved in assessment, prescription, monitoring and follow-up of people using Assistive Technology
- ☐ I am/ have been involved in assessment, monitoring and follow-up of people living with dementia
- ☐ I am/ have been employed as an occupational therapist
- ☐ Other

How long have you been qualified as a occupational therapist (in years, months)?

In which region/ country do you work?

Are you employed by NHS/ Social Services/ Private Organisation? Please give details:

What is your agenda for change banding (NHS) or equivalent?

Please describe your practice setting?

How do people access your service?

Please describe your work role in relation to assistive technology and/ or working with people with dementia.

I have completed training that relates to the provision of Assistive Technology

- Yes
- No

Please provide details of training provider and content of training.

Are you involved in the research or development of assistive technology for people with dementia? Please give details:

Section 2: Assessment

Is your clinical practice informed by use of a theory or model?

- ☐ Yes
- ☐ No

Is this

- ☐ MOHO
- ☐ CMOP-E
- ☐ Claudia Allen
- ☐ Model of Human Activity Assistive Technology (HAAT)
- ☐ Framework for Modelling the Selection of Assistive Technology
- ☐ Matching Person and Technology (MPT)
- ☐ Occupational Therapy Intervention Process Model (OTIPM)
- ☐ Other

Please give details of the other model you use to guide practice.

Please explain how your choice of model/theory benefits practice?

Are there aspects of practice which this model/ theory does not consider?

When carrying out needs assessment with a person living with dementia, which assessment tools do you use?

- [ ] AMPS
- [ ] COPM
- [ ] Claudia Allen/ LACLS
- [ ] MOHOST
- [ ] HAAT
- [ ] MMSE
- [ ] MEAMS
- [ ] Non-validated assessment tool
- [ ] Other

Please specify which other assessment tool you use in practice.

Do you use other assessment tools specific to certain client groups, interventions or needs? *Optional*

- ○ Yes
- ○ No

Which tools do you use?

Are there characteristics or circumstances which are difficult to assess using available assessment tools? e.g., clients with dementia and sensory difficulties, language differences, ability to operate assistive technology etc.

- Yes
- No

Please give details.

How do you currently manage this?

In circumstances where the person with dementia, and their caregiver or family member present different views of their assistive technology needs, how do you proceed?

How do you decide when re-assessment should take place?

Is there anything you feel is missing from this section? Or anything else you would like to mention regarding assessment processes?

Section 3: Intervention

Once you have undertaken needs assessment, how do you prioritise interventions?

- Focus on risk reduction
- Focus on priorities identified by the person
- Focus on priorities identified by the caregiver
- Focus on Autonomy/ Independence
- Other

If "other", please give details:

Please give reasons for your answer.

Where an appropriate intervention is not easily identifiable, how do you proceed? Which resources do you access to help you identify an appropriate intervention?

Are there any restrictions to the range of interventions you consider in your practice;

(please check all that apply)

- ☐ No restrictions
- ☐ Availability
- ☐ Cost
- ☐ Knowledge of range of interventions
- ☐ Limited services to support/ maintain interventions
- ☐ Service policies
- ☐ Other

Please give details for your answer.

Are you able to access a range of assistive technology interventions which meet the needs of people with whom you are working?

- ○ Yes
- ○ No

Please provide details regarding the assistive technology you are/ aren't able to access

Do you feel you have a good understanding of assistive technology interventions which

are available?

- Yes
- No

How did you obtain this knowledge? How do you keep this knowledge up to date?

What would help you to expand your knowledge in this field?

Do you feel there are instances where assistive technology interventions may be appropriate, but are not considered?

- Yes
- No

Please explain your answer.

Is there anything you feel is missing from this section? Or anything else you would like to

mention regarding assistive technology interventions?

Section 4: Sharing success

What supports you to develop expertise in this field?

What skills, attributes or experiences assist you in providing assistive technology interventions for people with dementia?

Do you feel additional support is required to improve the effectiveness of assistive technology services?

If you feel additional support is required, what might this look like?

How do the systems or processes within your organisation support your practice?

What helps to focus everyone in your workplace on the same goals or outcomes for AT service delivery?

What are the goals or outcomes for your team, in relation to assitive technology service delivery ?

In what way do the culture and working practices within your organisation support your practice?

Which organisations or professions provide support to help you to make a difference in this area? Please explain your answer.

Is there anything else you would like to say about the ways in which the characteristics of your practice environment support or restrict the delivery of effective assistive technology services for people with dementia?

Next Stage

Once we have collated the responses to this survey we aim to use this data to inform future research and service development, together with people who are interested in assistive technology for people with dementia. We anticipate that this will involve short interviews or focus groups in spring 2022. If you would like to be contacted regarding the next stage of the research please provide your contact details below. Your contact details will only be used to email you about this research. Providing contact details does not oblige you to participate in any further research.

Name

Email address

Final page

Thank you for completing this survey.
